# Nonsurgical Clinical Management of Periapical Lesions Using Calcium Hydroxide-Iodoform-Silicon-Oil Paste

**DOI:** 10.1155/2018/8198795

**Published:** 2018-02-12

**Authors:** Qusai Al Khasawnah, Fathi Hassan, Deeksha Malhan, Markus Engelhardt, Diaa Eldin S. Daghma, Dima Obidat, Katrin S. Lips, Thaqif El Khassawna, Christian Heiss

**Affiliations:** ^1^Experimental Trauma Surgery, Faculty of Medicine, Justus-Liebig University of Giessen, Giessen, Germany; ^2^Center of Dental Implants, Jordan German Dental Institute (JGDI), Amman, Jordan; ^3^Department of Trauma, Hand and Reconstructive Surgery, University Hospital of Giessen-Marburg, Giessen, Germany

## Abstract

**Background:**

The study aim is to avoid tooth extraction by nonsurgical treatment of periapical lesion. It assesses healing progress in response to calcium hydroxide-iodoform-silicon oil paste (CHISP). Numeric Pain Rating Scale was used to validate the approach. Furthermore, CHISP was used to treat cystic lesions secondary to posttraumatic avulsion of permanent teeth.

**Materials and Methods:**

Over 200 patients with radicular cysts were treated with CHISP through the root canal. Radiographs were used to verify lesion size and position, ensure correct delivery to the site, and monitor the progress of bone healing in the lesion area. Ten males and 10 females were randomly selected for statistical assessment.

**Results:**

No severe pain, complications, or failure in cyst healing was reported. Complete healing was achieved in an average of 75 days. Furthermore, healing of radicular cyst secondary to posttraumatic tooth avulsion was successful.

**Conclusion:**

CHISP indicated an antiseptic effect, which enhanced and shortened healing time of periapical lesions. The less invasive procedure avoids tooth extraction and reduces bone resorption. Cyst management with CHISP can remedy failed root canal treatments. The results show a bone regenerative capacity of CHISP suggested in first rapid phase and a second slow phase.

## 1. Introduction

Periapical lesion results from serious inflammatory response to microorganisms around the tooth root and the root canal [1]. Periapical lesions could perforate into the oral cavity affecting hard tissue or maxillary sinus. The infection around the root and tooth leads to bone resorption caused by local osteomyelitis [2]. Furthermore, cellulitis in soft tissue causing swelling in the face is a common symptom of severe local jawbone osteomyelitis. Traumatic injuries of teeth can cause granuloma or cysts associated with periapical lesions.

Granulomas are composed usually of solid soft tissue, while cysts are semisolid tissue surrounded by epithelium [3]. Radiographs show lesions structure as unilocular, lucent, round, or pear shaped contoured by a thin rim of cortical bone [4]. The incidence of cysts formation is between 6 and 55% in small lesions and a 100% with lesions larger than 20 mm [2]. On the other hand, granulomas occurrence ranges between 9.3 and 87.1%, where abscesses formation rate is between 28.7 and 70.07% [4].

Epithelial proliferation and other molecular mechanisms can cause lesion formation. Nonetheless, by-products of microorganisms, which lead to osmotic fluid accumulation in the lumen, are the most common cause of periapical lesions [5]. Therefore, eliminating microorganisms can release the hydrostatic pressure resulting from osmotic fluid and minimize the effect of periapical lesions on the tooth.

Management of infection-caused periapical lesions is a two-step process. Firstly, antibacterial treatment is performed using antibiotics (e.g., metronidazole, ciprofloxacin, and minocycline), chemical irrigation, and disinfectants (i.e., calcium hydroxide). Despite its wide use as disinfectant of the root canal system, calcium hydroxide is not effective as root canal dressing [2, 6, 7]. Secondly, releasing the hydrostatic pressure is detrimental and can be achieved by decompression, aspiration, and aspiration irrigation [8–10].

Forms of calcium hydroxide-iodoform-silicon-oil paste (CHISP) are commercially available for root canal treatment. Vitapex® is commercial nontoxic product containing a viscous mix of iodoform (40.4%), calcium hydroxide (30%), and silicone oil (22.4%). The paste is administered through a syringe with disposable tips. In clinical practice, commercially available CHISP is recommended to manage apexification/apexogenesis procedures. The antiseptic properties of the paste support root development, while tooth pulp heals [11]. Properties and mode of action of the calcium hydroxide as the main component of the paste were fully reviewed [12].

In the last decade, two clinical case studies reported unintentional extrusion of CHISP beyond the root and into the periapical lesions. Furthermore, both studies did not report any complications or side effects resulting from CHISP placement in bone [13, 14]. Interestingly, a rat model study revealed bone regeneration capacity of CHISP. The study utilized Vitapex to treat periapical lesions in rats and showed enhanced BMP-2 expression both histologically and molecularly [15].

The lack of complications in clinical cases and the promoting effect on healing in the experimental data encouraged us to conduct a clinical trial on systematic use of CHISP to treat periapical lesions.

The present study aimed to utilize CHISP in the nonsurgical administration protocol to treat periapical lesions. CHISP is favored due to its antiseptic and bone regenerative properties.

The study hypothesized that the use of CHISP, circumventing a surgical procedure, results in easier management of periapical lesions, faster healing due to enhanced bone formation, and a reduced posttreatment pain management. Furthermore, the use of CHISP to manage failed root canal treatments and posttraumatic tooth avulsion shows potential to avoid tooth loss.

## 2. Materials and Methods

Over 200 patients with one or more periapical lesion were treated between 2013 and 2015. Randomly, 20 patients (10 females and 10 males, with 29 lesions) were analyzed for the present study. Population for statistical analysis was randomized for each gender. Clinical examination revealed periapical cystic lesion ranging between 4 and 8 mm either due to the pulpits or due to previous root canal treatment.

The study population was recruited from patients of the Jordan German Dental Institute (JGDI), Amman, Jordan, during two years. Each patient, who was in need of periapical lesion treatment, whether for the first time or after failed root canal treatment, was offered participation in the study. Patients were charged for the treatment to keep the cost from affecting their decision. The patients committed to attend the periodic follow-up by participating in the study. No financial compensations were offered and the patients maintained the right to quit the study without giving any reason. Prior to treatment, each patient provided written, informed consent to participate in the study. The clinical trial was approved by the ethics committee of the JGDI under EA-number 7/2013.

### 2.1. Nonsurgical Approach to Deliver the Paste to the Lesion

The patients were treated with CHISP (either Vitapex, NEO Dental Inc., Moringen, Germany, or Metapex®, MetaBiomed, Chungbuk, Korea) under local anesthesia (2% Xylocaine Dental with epinephrine 1 : 50,000, Novocol Pharmaceutical of Canada, Inc., Ontario, Canada).

**Figure 1:**
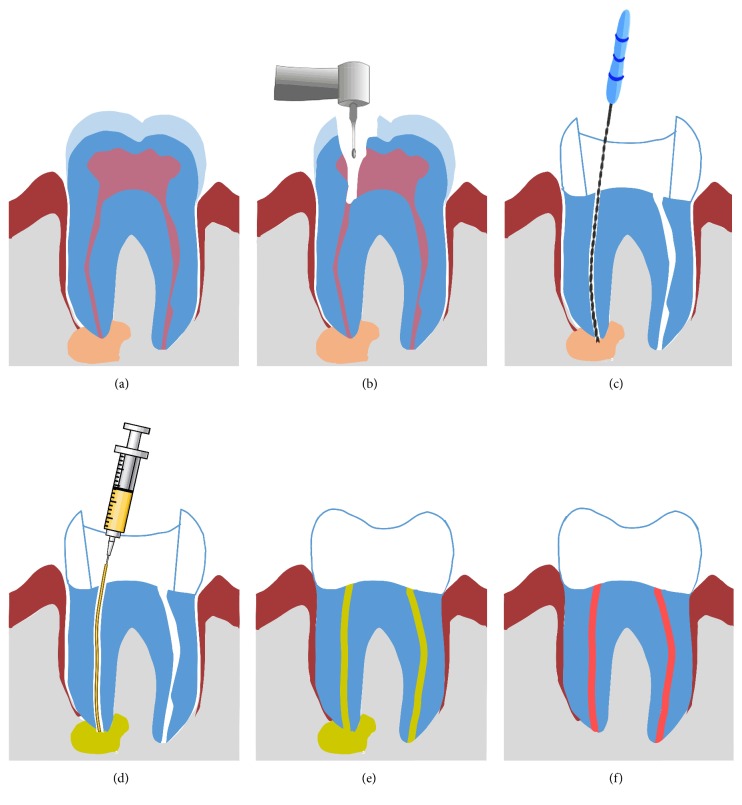
Schematic drawing showing a step-by-step procedure of cyst treatment through the root canal using CHISP. (a) Assessment of cyst size and position using radiographs. (b) Tooth is opened to access root canal. (c) File implementation to reach infected area and cyst. (d) CHISP is injected through the drilled root canal until the cyst is filled; filling is assessed by intraoperative radiograph. (e) The filling enhances tissue healing while resorbing, allowing bone formation. (f) Complete healing and bone formation, no radiolucency is seen near the root.

After periapical lesions were verified by radiographs, patients were informed about the new treatment option. Upon consent, patients received a nonsurgical root canal treatment. Briefly, pulp was extirpated and cleaned; then the canals of the infected teeth were opened and shaped using rotary and manual files. To relief pressure and establish drainage through the canal, the file should be carried up about 1-2 mm beyond the apical foramen (Figures 1(a)–1(c)). Negative pressure was created using 22 G needle fixed to a high-volume suction aspirator. The needle was inserted into the root canal and activated for 3–5 minutes. Occasionally, to eliminate the cystic fluid in the periapical lesion buccal-palatal aspiration approach is required. A 12 G needle was used to penetrate the mucosa and aspirate the cystic fluid. The prepared root canals were then irrigated with 5% sodium hypochlorite to eliminate debris and to disinfect the canal. The access canal was then closed with a small cotton pellet to maintain drainage until second session.

After 24–28 hours, the created root canal was washed with 5% sodium hypochlorite. Subsequently, the tip of the CHISP syringe was introduced as close as possible to the periapical lesions. Then, the paste was injected through the canal until the lesion was adequately filled (Figure 1(d)). The position of filling was controlled intraoperatively using radiography.

### 2.2. Posttreatment Instructions and Follow-Up

Patients were instructed to refrain from eating for one hour and to cool the area for 24 hours. Solid food was avoided for 48 hours and Clindamycin (Dalacin C 300 mg, Pfizer Corporation, Vienna, Austria) was prescribed. Healing was monitored by radiological follow-up: 10, 30, 60, and 120 days posttreatment. During the first week, dentists communicated with the patient on daily bases. Beside pain scale, the dentists asked about allergic reaction, complication, and any possible side effects. Follow-up data was logged for further analysis. In few cases, radiographs showed paste detachment from the apex. In such cases, permanent obturation of the canal was performed using Pulpdent® (Pulpdent Watertown, MA) as root canal sealer. Pulpdent is tissue compatible, bacteriostatic, and radiopaque. The canal space was filled with Gutta-percha (VDM, Munich, Germany). Sealing the canals is critical to prevent bacterial reinfection. Finally, composite material was used for the permanent filling of the tooth cavity. Nevertheless, welfare of patients is utmost priority that is judged by posttreatment pain.

### 2.3. Pain Assessment after Periapical Lesion Treatment with CHISP

A numerical pain assessment scale was followed as described previously [16]. The ten-point Numeric Pain Rating Scale (NPRS) ranges from no pain, 0 point, until extreme pain, 10 points. Patients were prescribed Brufen 600 mg (Abbott, Vienna, Austria) and instructed to take it only when needed. Furthermore, a daily checkup for the first 4 days was performed, where patients were asked to describe the pain with one word: none, mild, moderate, strong, and extreme. Patients were reminded to fill the pain points scale form every day. Moreover, patients were instructed to call a direct line at any time if the pain management did not result in pain relief.

### 2.4. Statistical Analysis

Correlation of lesion size with healing was performed using bivariate analysis and spearman's rho test for nonparametric correlations. Lesion size correlation to gender is depicted as box plots. Bar graphs demonstrate frequency analysis performed using chi-square for lesions count per patient, healing time, and pain scale. Statistical analysis was performed using IBM SPSS software V. 21.0 (CA, USA), and significance cutoff was considered *p* ≤ 0.05 and highlighted as asterisks. Patient cases in the final analysis of this study were randomly selected using SPSS. The option “select cases,” followed by random sample of cases, was applied after filtering according to gender option. Ten cases were chosen to represent each gender. Sample size was determined by power analysis using GPower software [17]. Dentist identity, patient history, and paste manufacturer information were blinded for the analysis.

## 3. Results

The study shows an alternative to surgical cyst treatment which results in bone regeneration as early as 40 days posttreatment.

Nonsurgical treatment was carried out for over 200 patients suffering from periapical lesion. To eliminate bias and compare gender variability and pain indications, a population of ten patients of each gender was randomly selected. The treatment encompassed a deliberate injection of commercially available CHISP through the root canal into the lesion. The healing mode value was 60 days in both genders. Complete healing was determined by lack of radiolucency in radiographs. Pain indicator ranged from none to moderate pain descriptively and from 0 to 4 points according to pain scale.

### 3.1. No Gender-Related Differences in Lesion Size and Frequency

**Figure 2:**
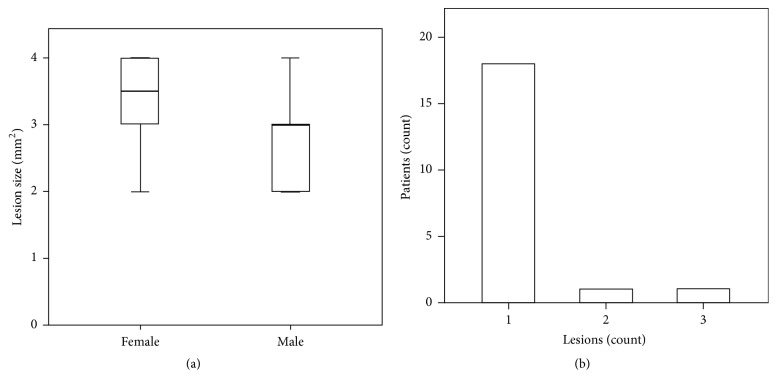
Lesion size and frequency did not exhibit gender variations. (a) Lesions size in female patients was not significantly different when compared to male patients. (b) Only 10% of patients suffered from more than one lesion.

Radiolucency of periapical lesions is the evaluation criteria of size and position of the cyst. The size of the lesions for the randomly selected patient population ranged from 2 to 4 mm (Figure 2(a)). Nonetheless, out of the stem population of 200 patients, lesions of about 10 mm in size healed with a successful bone formation within 120 days. Lesion size did not show any gender variation (Figure 2(a)) [gender; mean ± SD; maximum : minimum, F; 3.3 ± 0.82; 4 : 2, M; 2.7 ± 0.67; 2 : 2]. Furthermore, the frequency of lesions per patient ranged between one and three lesions. The majority of patients showed one lesion in the radiographs, and the frequency of two and three lesions was 5% each (Figure 2(b)).

### 3.2. CHISP Retention in the Lesion Is Proportional to Bone Healing Progression

The procedure was fast and patients of neither gender reflected allergic reaction. The follow-up period was longer than two years for the first cases and no failure or recurrent lesion formation was reported in any case.

**Figure 3:**
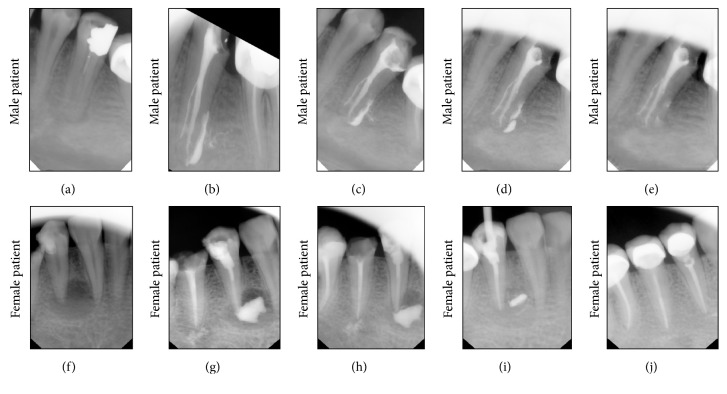
Randomly selected cases to represent healing after CHISP injection into the lesion. The upper panel shows the effect of CHISP leading to gradual healing of the lesion in male patient, while the lower panel shows the healing in female patient. (a and f) Identification of lesion under radiolucency criteria. (b and g) Lesion filling with CHISP either in full as in male patient or partially as in female patient. (c and h) Follow-up after 10 days exhibits a clear degradation of the paste and lesser radiolucency in the lesion. (d and i) 60-day posttreatment, the bone quality is improved proportionally to the material retention. (e and j) Full resorption of CHISP with complete bony healing.

Smaller and larger lesions were treated with the no significant difference in average healing time. Nonetheless, Spearman's rho correlation showed a trend (*p* = 0.055) that the time of healing is longer for larger lesions [parameter; mean ± SD; maximum : minimum, lesion size; 3.0 ± 0.79; 4 : 2, healing time; 75.78 ± 23.8; 120 : 40]. Resorption of the paste along was proportional to the regeneration of the bone. No single case required revision and reinjection. Representative cases of each gender were randomly selected out of the 10 patients to avoid bias (Figure 3). Interestingly, female patient depicted case shows that the lesion must not be filled completely to reach satisfying results (Figures 3(f)–3(j)). Gradual degradation of CHISP occurred in correlation to the newly formed bone.

### 3.3. Complete Healing Assessed by Lack of Radiolucency

Healing of periapical lesions was qualitatively examined by radiographs. One male patient showed a complete healing of a 3 mm large lesion after 40 days of treatment. However, 35% of lesions healed after 60 days of treatment (40% of female patients and 30% of in male patients). Interestingly, 30% of lesions healed at 90 days posttreatment in females compared to none in males.

**Figure 4:**
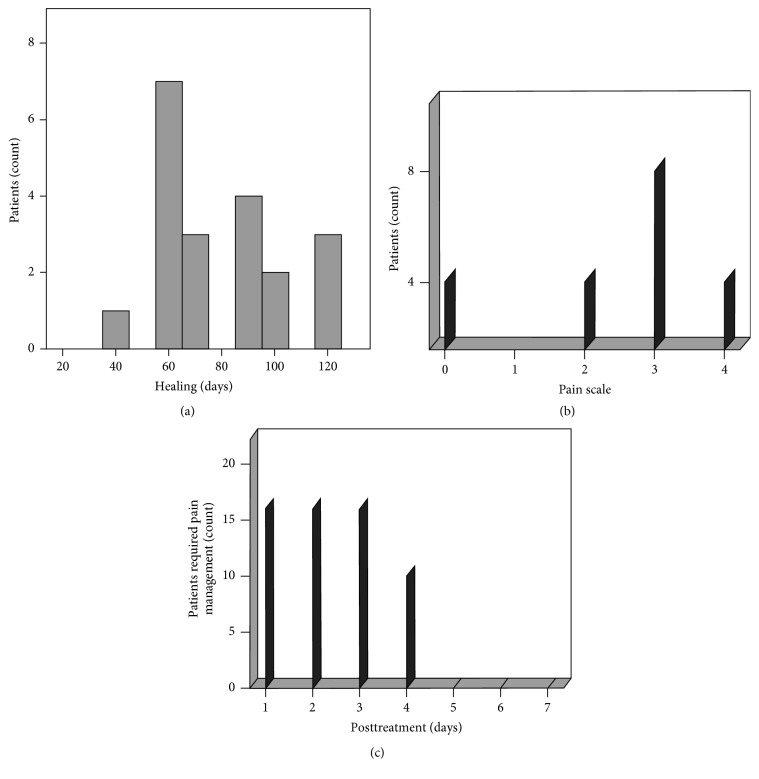
Healing of periapical lesion is achieved in 60 days with mild to moderate pain. (a) Patients showed most frequent healing after 60 days of treatment; the second frequent complete healing was after 90 days. Longest healing time was 120 days posttreatment. (b) Mild pain was described by 40% patients after treatment. However, lesser pain description and scale were reported of about 40%. Moderate pain with a scale of four was described by 20% of patients. (c) Analgesics were required by 80% of the patients in the first 3 days. After 5 days none of the patients needed a medication for pain relief.

However, in general the longest healing time in both genders was 120 days posttreatment (Figure 4(a)). Complete healing at 60 days posttreatment was most frequent (50% of the cases), followed by 90 days and 120 days. The average healing time was 75.78 days ± 23.8 days.

### 3.4. Moderate Pain Associated with the CHISP Procedure

Patient tolerance to pain is related to subjective estimation. However, the descriptive and scale assessment used in this study can indicate the expected pain caused by the CHISP treatment of lesions.

Around 20% of the patients did not require pain management drugs after treatment (0 points). However, over 40% described their pain as mild (3 points). Around 20% described the pain as moderate and scaled it as 4 out of 10 points. The remaining 20 percent described the pain as mild and scaled it at 2 points (Figure 4(b)). However, only 20% of all patients did not request pain management drugs. Interestingly, pain complaints did not last longer than 7 days, regardless of pain management. However, 80% of the patients started pain management drugs after the treatment; after day three, 50% only required the medication that was not required beyond day four (Figure 4(c)).

## 4. Discussion

Periapical lesion is an inflammatory process affecting soft and hard tissues surrounding the tooth. The inflammation is associated with the loss of supporting bone, bleeding on probing and suppuration. Necrosis of the pulp found suitable environment for microorganisms to release toxins into periapical tissue. This secretion leads to inflammatory reaction, which is associated with periapical lesion formation.

A systemic literature review by Froum 2011 [18] showed that the ideal management of lesions should focus on infection control of the lesion and regeneration of lost support. The treatment options for large periapical lesions range from conventional nonsurgical root canal therapy to surgical interventions [4]. Nonsurgical root canal treatment should always be the first choice in cases of nonvital teeth with infected root canals. Elimination of bacteria from the root canal is the key of periapical lesions treatment [13].

Vitapex, Metapex, and Tegapex® are commercially available premixed calcium hydroxide-iodoform-silicon-oil paste. The products are used as a temporary or permanent root canal filling material after pulpectomy. The paste has excellent antibacterial and bacteriostatic properties and promotes apexification and apexogenesis.

Calcium hydroxide has ionic effect observed by chemical dissociation into calcium and hydroxyl ions. Calcium and hydroxyl ions have antimicrobial effects and induce mineralization. Calcium hydroxide stimulates “blast” cells aiding apexogenesis and its high pH neutralizes endotoxins produced by anaerobic bacteria. Hydroxyl ions act on the cytoplasmic membrane of bacteria and it enhances tissue enzymes activity such as alkaline phosphatase which plays a role of extending roots and apical closure [12, 13, 15]. Iodoform has bacteriostatic property by releasing free iodine. Thereby, iodine eliminates the infection of root canal and periapical tissue by precipitating protein and oxidizes essential enzymes [19]. Iodoform also enhances radiopacity for better visualization. Silicone oil is a lubricant, which ensures complete coating of canal walls and solubilizes calcium hydroxide to remain active in root canal.

Recently, CHISP were reported to induce bone formation of apical periodontitis and periapical bone regeneration* in vivo* due to expression of BMP-2 in rats [15]. Furthermore, Singh et al. concluded that extrusion of Metapex unintentionally into periapical lesion showed no negative effects or complications [13, 14]. Both studies encouraged us to start the presented clinical trial.

The present study provides a clear evidence of the enhanced healing of lesions using CHISP. The healing time in the studied cases was between 40 and 120 days (Figure 4(a)). Thereby, the nonsurgical procedure is one-sixth to one-fourth of the time reported for the conventional treatment of 12 months [7, 13] and 24 months [1], respectively.

The results showed that the material degradation is qualitatively faster in the first ten days in comparison with the 60-day radiograph. This observation suggests that the paste has a rapid degradation in the first phase, which becomes slow after 10 days. However, such observation can only be confirmed in Cone beam computed tomography three-dimensional imaging with quantitative analysis.

**Figure 5:**
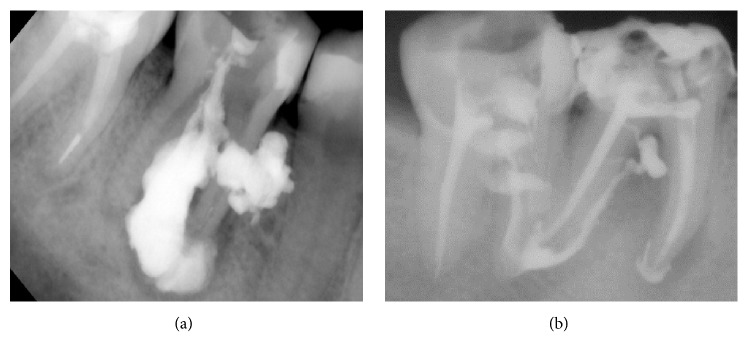
CHISP management can remedy failed root canal treatment of periapical lesion. Recurrence of lesion after failed conventional root canal treatment is high. CHISP offers a nonsurgical treatment with high success rate. (a) Radiograph showing lesions filled with the CHISP through the prepared root canal and buccal-palatal approach. (b) CHISP can be used for smaller lesions.

Furthermore, failure of conventional treatment requires the resort to more invasive treatment and can lead to tooth loss, bone grafts, and eventually dental implant. Nonetheless, radiographs showed the successful delivery of CHISP to the periapical lesions after failed root canal treatment. The lesion can be reached by buccal-palatal approach (Figure 5(a)) or through reopening the canal (Figure 5(b)). Higher resorption capability makes CHISP a suitable filling to treat failed cases of conventional method. Moreover, targeted delivery of CHISP is beneficial when periapical lesions occur in close vicinity of vital cells.

Posttraumatic dental injury is a known etiology of periapical lesions [20]. In some cases, neighboring teeth suffer an additional and unnoticed injury, which can also result in periapical lesion formation.

The current study showed a promising management of posttraumatic tooth avulsion using CHISP. The paste was used to treat a trauma injury in the lower central incisors. Teeth were avulsed and kept as soon as possible in cold cow milk. The patient was admitted two hours after the trauma suffering from bleeding, tissue swelling, and pain.

**Figure 6:**
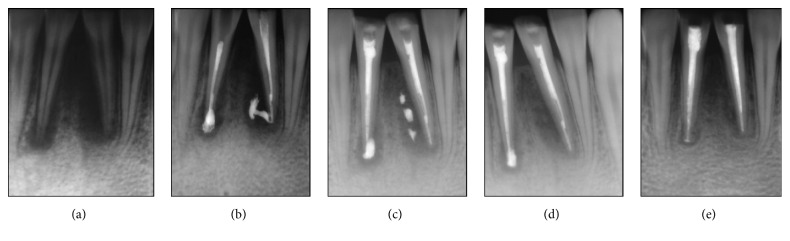
Bone defect and inflammatory resorption resulting from traumatic injury can be treated with CHISP. Inflammatory resorption at the root secondary to granulomas and infection in the pulpal space after avulsion were successfully treated using CHISP. (a) Preoperative radiograph of a clinical case of traumatic dental injury. (b) Intraoperative radiograph showing the injected paste through the root canal. (c) 10 days posttreatment exhibits fast material degradation. (d) Significant reduction of gap size and clear formation of bony tissue around the root of injured teeth. (e) Healing improvement and paste degradation after 60 days.

Clinical examination of the lower jaw indicated mobile and badly injured gingival ligaments. The injury site was repeatedly washed with normal saline for sterility and better vision. The teeth were replaced and fixed by splinting using glass ionomer (DMG, Hamburg, Germany). Periapical X-ray showed enlargement in the periodontal ligaments and lamina dura (bundle bone) as well as affected pulp (Figure 6(a)). Pain management and anti-inflammatory treatment were then prescribed to the patient.

After three days, the patient complained from pain and teeth mobility, although no swelling was present. Therefore, root canal treatment was performed. CHISP (Vitapex) was injected in the canals and the defect gaps around the roots (Figure 6(b)). The access cavity was closed with small cotton pellets and long-lasting light cure temporary filling. After 10 days, the teeth mobility was insignificant and the patient did not complain from pain (Figure 6(c)). After one-month significant improvement in the defect healing was seen (Figure 6(d)). Two months posttreatment vast degradation CHISP correlated to enhanced bone regeneration around the tooth apex (Figure 6(e)). Therefore, a permanent obturation of canals and permanent filling of the teeth cavity were performed.

## 5. Conclusion

The use of calcium hydroxide-iodoform-silicon-oil paste as nonsurgical approach for treatment of periapical lesions showed a high success rate. The bone regenerative effects are detrimental to the success of the treatment. This effect was confirmed by applying CHISP to regenerate bone around the roots of avulsed tooth posttrauma. Healing of periapical lesion within 2 months with mild to moderate pain indication is crucial properties when comparing CHISP to conventional treatment.

Although material retention analysis requires 3D image set, the availability of this information can provide better understanding to the bone-material interaction. Furthermore, the regenerative effect of CHISP can be examined in nondentistry related indications in orthopedic and trauma surgery field.

## Additional Points

*Highlights*. (i) Nonsurgical approach for periapical lesion treatment. (ii) Successful cyst closure by calcium hydroxide-iodoform-silicon-oil paste. (iii) Tooth preservation by bone regenerative and anti-infectious capacity. (iv) Average healing of 75 days of periapical lesions with 4 mm average size.
